# An evaluation of Massachusetts’ flavored tobacco restriction one year after policy implementation: Is it promoting equity?

**DOI:** 10.1057/s41271-026-00633-w

**Published:** 2026-05-06

**Authors:** Jill M. Singer, Jeffrey J. Wing, Elizabeth G. Klein, Micah L. Berman, Megan E. Roberts

**Affiliations:** 1 College of Public Health, The Ohio State University, Columbus, OH United States; 2 Moritz College of Law, The Ohio State University, Columbus, OH United States

**Keywords:** Tobacco, Cigarettes, Health equity, Policy, Flavor restrictions

## Abstract

In the United States certain groups, such as African Americans (AA) and sexual minorities (SM) have disproportionately high rates of flavored tobacco use. Policies restricting flavored tobacco may especially benefit these groups and lead to an equitable reduction in tobacco use. This study used Behavioral Risk Factor Surveillance System data from Massachusetts and Connecticut (control state) to evaluate changes in cigarette use before and after Massachusetts’ flavor restriction policy was implemented. Difference-in-difference (DID) was used to evaluate the overall impact and difference-in-difference-in-difference (DDD) was used to evaluate the impact on AAs and SMs. There were no significant differences in the change in cigarette use for adults overall or for AA adults in Massachusetts compared to Connecticut. Among SMs, Connecticut had a greater decrease in cigarette use compared to Massachusetts. As more data become available, it will be crucial to monitor the longer-term impact of the policy and any differences in policy impact among groups.

**Supplementary Information:**

The online version contains supplementary material available at 10.1057/s41271-026-00633-w.

## Introduction

The tobacco industry has a long history of targeting its harmful products to historically marginalized groups. One of the most egregious examples is the industry’s targeting of menthol cigarettes to the African American (AA) community [1]. Tobacco companies used popular icons as spokespersons, placed advertisements in magazines with predominantly AA audiences, and contributed money to civil rights organizations and AA community organizations [2–5]. These marketing strategies effectively increased menthol cigarette smoking among *everyone*, but especially among African Americans. Non-Hispanic AAs who smoke are more likely to smoke menthol cigarettes compared to non-Hispanic Whites who smoke [6]. In 2019, 85% of AAs who smoked cigarettes reported currently smoking menthol cigarettes; an even higher proportion (90%) reported that they initiated use with menthol cigarettes [7, 8]. In contrast, less than half of non-Hispanic Whites who smoked initiated use with menthol cigarettes and menthol cigarette sales made up 37% of all cigarette sales in 2019 [7, 9].

Sexual minorities are another historically marginalized group that has been targeted by the tobacco industry [10, 11]. Similar to the targeting of the AA community, the tobacco industry placed models on their advertisements that reflected their target population and placed the advertisements in places where a high proportion of the people exposed to the advertising were sexual minorities [10, 12]. The prevalence of menthol smoking is higher among sexual minority adults compared to heterosexual adults overall and among those who currently smoke [13]. Within this community, there is variation in menthol cigarette use. Bisexual women report the highest prevalence of current menthol cigarette use (18.27%) followed by lesbian women (14.83%), gay men (11.33%), bisexual men (8.65%), heterosexual men (7.56%), and heterosexual women (6.98%) [14]. Additionally, those who smoke menthol and identify as sexual minorities have lower odds of reporting intention to quit smoking for good compared to those who smoke menthol and identify as heterosexual [15].

In summary, flavored tobacco products (menthol cigarettes) contribute considerably to tobacco use disparities and, ultimately, tobacco-related health disparities [16, 17]. Therefore, flavored tobacco restriction (FTR) policies have the potential to most benefit historically marginalized groups with higher rates of flavored tobacco use, and thus improve health equity [18]. FTRs are a relatively new tobacco control policy, but have been adopted in a number of jurisdictions [19]. Some FTRs are narrow and only apply to certain products (e-cigarettes), while others are comprehensive and apply to all types of tobacco products. Many policies are implemented at the local level (county, city), but some states have begun adopting FTRs as well. In June 2020, Massachusetts became the first state to implement a comprehensive FTR.

In 2014, municipalities in Massachusetts began restricting the sale of flavored tobacco products, modeling their policies after a restriction in Providence, Rhode Island [20, 21]. In 2019, a sudden rise in e-cigarette or vaping use-associated lung injury (EVALI) led the then governor, Charlie Baker, to ban the sale of all vaping products for four months (September 2019–January 2020) [22]. During this four-month ban, Governor Baker signed into law *An Act Modernizing Tobacco Control* [23]; this law prohibited the sale of all flavored tobacco products and was the first statewide comprehensive FTR in the country. The restriction on flavored e-cigarettes was effective immediately (November 2019) and the full policy (restricting all types of flavored tobacco) was implemented on 1 June, 2020.

The purpose of this study was to evaluate the impact of Massachusetts’ FTR on the prevalence of cigarette use both overall and among African Americans and sexual minorities (SM), groups with higher rates of flavored tobacco use. We looked at the change in prevalence in Massachusetts before the policy was implemented and one year after the policy was implemented compared to Connecticut, where there are no FTRs. Our hypotheses were: 1) the FTR in Massachusetts would lead to a greater decrease in cigarette use among adults in Massachusetts relative to Connecticut and 2) AA and SM groups in Massachusetts would benefit more from the policy (have a greater decrease in tobacco use) than their counterparts in Connecticut.

## Data and Methods

### Study design

This study was a secondary data analysis evaluating the impact of Massachusetts’ FTR on the prevalence of cigarette use among adults overall and among African Americans and sexual minorities. Analyses used a difference-in-difference (DID) approach, which allowed us to compare the trajectory of tobacco use in Massachusetts before and after the policy was implemented to a counterfactual where no policy was implemented. Connecticut was chosen as the counterfactual (control state) for the analysis because of the similarities between the two states, including demographics [24, 25], smoking prevalence [26, 27], cigarette prices [28], and tobacco control policy [29, 30]. Specifically, for tobacco control policies the year after policy implementation, the American Lung Association (ALA) gave Massachusetts and Connecticut the same grades on *Tobacco Prevention and Cessation Funding, Tobacco Taxes, and Access to Cessation Services*, but Massachusetts received an “A”—indicating excellent tobacco control policies—for *Flavored Tobacco Products* while Connecticut received an “F”—indicating inadequate tobacco control policies. Massachusetts also received a better grade on *Smokefree Air* compared to Connecticut (A vs. C) [29, 30].

### Data source

This study used data from the Behavioral Risk Factor Surveillance System (BRFSS), an annual, state-based, telephone survey conducted in the United States and its territories that collects data on adults’ (age 18 +) health-related behaviors [31]. For the current analysis, data from 2015 to 2019 state BRFSS surveys in Massachusetts and Connecticut were used to examine the prevalence of cigarette use and trends before the FTR in Massachusetts was implemented (pre-policy timepoints). BRFSS data from 2021 surveys in Massachusetts and Connecticut were used for the post-policy timepoint (Supplementary Material Table 1). Overall survey response rates ranged from 32.8% to 50.6%. Missing data were not statistically imputed. The university’s institutional review board (IRB) determined this study was Not Human Subjects Research (NHSR) and no IRB approval was needed.

### Measures

#### Demographic measures

Demographic measures for *race* and *sexual identity* were used to assess how the policy impacts groups who have higher rates of flavored tobacco product use. Race was dichotomized as a person identifying as African American or Black and not Hispanic versus a person identifying as any other race or Hispanic. For sexual identity, a person was coded as a sexual minority if they considered themselves to be something other than straight or heterosexual versus not a sexual minority if they considered themselves to be straight or heterosexual.

#### Outcome measure

The outcome of interest was current cigarette use. Respondents were coded as currently using cigarettes if they had smoked 100 cigarettes in their life and currently smoked cigarettes some days or all days; they were coded as not currently using if they had never tried cigarettes, never smoked 100 cigarettes in their life, or did not currently smoke cigarettes [31].

### Analysis

To assess the impact of the policy overall, DID was used to evaluate the natural experiment occurring between Massachusetts’ FTR implementation and Connecticut’s lack of policy implementation. We used a log-binomial regression model that included the state (Massachusetts as treatment vs. Connecticut  as control), the policy period (pre-policy vs. post-policy implementation), and the interaction between the state and policy period. To assess the impact of the policy among African Americans and sexual minorities, we used a difference-in-difference-in-difference (DDD) approach. Separate log-binomial regression models were used for AA and SM groups.

Parallel trends were assessed using graphical checks of the pre-policy timepoints (2015–2019). The similarities described earlier between Massachusetts and Connecticut also support the assumption that changes between the two states after the FTR was implemented are a result of the policy and not of other underlying differences.

Analyses were conducted using Stata. All analyses were weighted and adjusted for survey design. An alpha level of 0.05 was used to determine statistical significance for the DID analysis and 0.10 for the DDD analysis.

## Results

### Overall adult trends

**Fig. 1:**
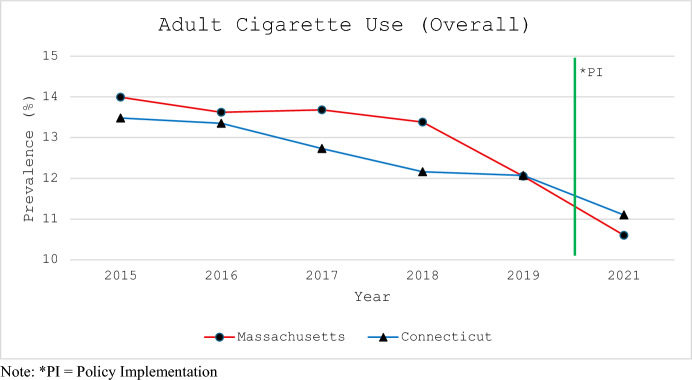
Prevalence of cigarette use among adults in Massachusetts and Connecticut, 2015–2021

Cigarette use in both Massachusetts and Connecticut decreased in the years preceding Massachusetts’ FTR. From 2015 to 2019, cigarette use decreased from 13.99% to 12.05% among Massachusetts adults and from 13.48% to 12.07% among Connecticut adults (Fig. 1). After implementation of Massachusetts’ FTR in June 2020, cigarette use continued decreasing in both states. In 2021, the prevalence of cigarette use was 10.61% among adults in Massachusetts and 11.10% among adults in Connecticut. The DID model suggests there was a slightly greater decrease in cigarette smoking in Massachusetts relative to Connecticut after policy implementation (Prevalence Ratio [PR]: 0.91, 95% Confidence Interval [CI]: 0.79, 1.05; *p* = 0.208); however, it was not statistically significant (Table 1).

**Table 1:** Prevalence ratios and 95% confidence intervals for difference-in-difference (DID) and difference-in-difference-in-difference (DDD) analysis

Tobacco Product	Identity	Overall PR^a^ (95% CI^b^)	Marginalized identity PR (95% CI)	Non-marginalized identity PR (95% CI)	P-value^c^
Cigarettes					
	Overall	0.91 (0.79, 1.05)	–	–	0.208
	African Americans	–	1.11 (0.66, 1.86)	0.89 (0.76, 1.03)	0.424
	Sexual minorities	**–**	**1.35 (0.88, 2.06)**	**0.86 (0.73, 1.00)**	**0.050**

^a^Prevalence ratio ^b^Confidence interval ^c^p-value from DID (overall) or DDD (African Americans and Sexual Minorities) interaction; Bold = *p*< 0.10

### Trends among AA groups

**Fig. 2:**
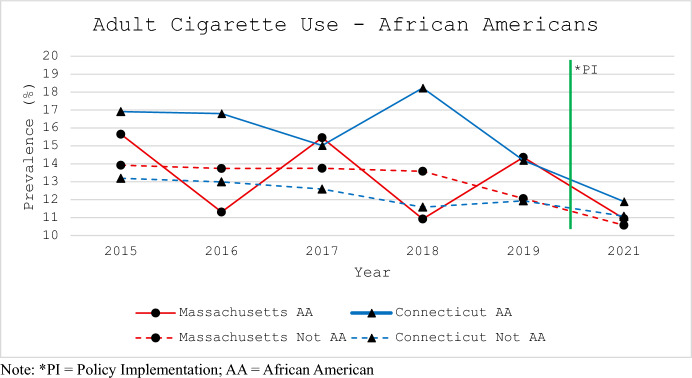
Prevalence of cigarette use among African American and non-African American adults in Massachusetts and Connecticut, 2015–2021

There was a higher prevalence of cigarette smoking among African Americans in both Massachusetts and Connecticut (15.65 and 16.91%, respectively) in 2015 compared to the general population. The prevalence among African Americans in Massachusetts fluctuated during the pre-policy period, ending at 14.36% in 2019 (Fig. 2). The prevalence also fluctuated in Connecticut; in 2019, the prevalence of cigarette use among African Americans was 14.19%. After implementation of the Massachusetts FTR, the prevalence of cigarette use among African Americans dropped to 10.94% in Massachusetts and 11.89% in Connecticut. The difference in the policy’s impact on African Americans (PR: 1.10, 95% CI: 0.66, 1.86) and non-African Americans (PR: 0.89, 95% CI: 0.76, 1.03) between Massachusetts and Connecticut was not statistically significant (*p* = 0.42).

### Trends among SM groups

**Fig. 3:**
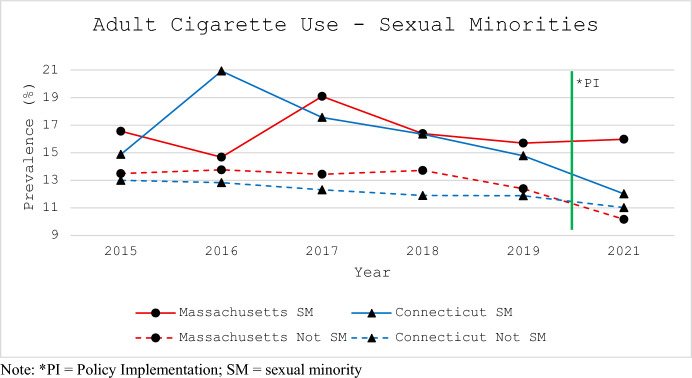
Prevalence of cigarette use among sexual minority and non-sexual minority adults in Massachusetts and Connecticut, 2015–2021

In 2015, 16.56% of sexual minorities in Massachusetts reported cigarette smoking compared to 15.70% in 2019 (Fig. 3). In Connecticut, the prevalence of cigarette smoking among sexual minorities was 14.88% in 2015 and was similar (14.78%) in 2019. In 2021, Massachusetts saw a slight increase in cigarette smoking among sexual minorities (15.98%), while Connecticut saw a decrease (12.02%). Similar to African Americans, the prevalence of cigarette smoking among sexual minorities was higher than that of the general population.

There was a statistically significant difference (*p* = 0.05) in the policy’s impact on sexual minority cigarette use (PR: 1.34, 95% CI: 0.88, 2.06) compared to non-sexual minority cigarette use (PR: 0.86, 95% CI: 0.73, 1.00) between Massachusetts and Connecticut. In Massachusetts, cigarette use among non-sexual minorities decreased more in Massachusetts after policy implementation compared to non-sexual minorities in Connecticut; however, cigarette use among sexual minorities in Massachusetts did not decrease more after policy implementation compared to sexual minorities in Connecticut. The results indicate that the policy was more beneficial (resulted in a greater decrease in smoking) for non-sexual minorities compared to sexual minorities in Massachusetts.

## Discussion

One year after Massachusetts’ FTR was implemented, there were no statistically significant changes in current cigarette use among Massachusetts adults overall (relative to Connecticut adults) or among Massachusetts African Americans (relative to Connecticut African Americans). There was evidence that the policy had an inequitable effect on sexual minority cigarette use. Specifically, the policy led to a 14% greater reduction in cigarette use in Massachusetts compared to Connecticut among *non*-sexual minorities but no greater decrease in cigarette use in Massachusetts compared to Connecticut among sexual minorities.

This study is among the first state-level evaluations of a FTR with a control group. The lack of impact overall aligns with the findings from an evaluation conducted in San Francisco that found no change in cigarette use after a menthol ban was implemented [32]; however, the sample comprised clients in a substance use disorder treatment program, not the general adult population. A separate study of San Francisco’s comprehensive FTR that included young adults found changes in tobacco use after policy implementation [33]; among adults ages 18–24, there was a significant decrease in cigarette use after policy implementation. However, among adults ages 25–34, there was no decrease in cigarette use after policy implementation. Importantly, neither study had a control group of participants who were not under an FTR. As cigarette use continues to decrease among adults [34, 35] it is important to have a control group so that the effects of that specific policy can be isolated from the underlying secular trends.

In a national sample that did compare prevalence between those under an FTR and a control group, there was a significantly lower prevalence of current tobacco use among those under a policy (20.55%) compared to those not under a policy (23.98%) [36]. However, this study included participants aged 15–36 years and did not disaggregate the type of tobacco products, making it hard to compare the study findings to our own. The present study adds to the mixed findings on whether FTRs decrease adult tobacco use.

When we disaggregated the data, we found evidence that Massachusetts’ policy had a significant impact on sexual minority cigarette use. Contrary to the original hypothesis, cigarette use among sexual minority adults in Massachusetts *increased* slightly after policy implementation, while cigarette use decreased among sexual minority adults in Connecticut. Although this study did not investigate potential reasons for this finding, it is possible that there were unique stressors to sexual minority adults in Massachusetts that resulted in increased cigarette smoking. Additionally, because of the higher prevalence of cigarette smoking (menthol and non-menthol) among sexual minorities compared to the general population [37] this policy may have nudged this group toward smoking non-menthol cigarettes instead of cessation. However, more work is needed to explore these possibilities and understand this result.

Although there were non-significant results for African American cigarette use, these null results are important to highlight. Menthol cigarettes are a driver of tobacco-related health disparities among African Americans [16, 17]. There are many who advocate for a menthol ban to reduce health inequities among African Americans [38, 39], and modeling and simulation studies predict menthol bans would save hundreds of thousands of lives, many of them African American, over the span of a few decades [40, 41]. Yet, in the present study, there was no indication that Massachusetts’ FTR reduced cigarette smoking among African American adults.

One potential reason for the unexpected effects is the continued access to menthol cigarettes in bordering states. A study among Massachusetts adults who smoked menthol cigarettes found two thirds of respondents accessed menthol cigarettes in another state after the FTR was implemented [42]. Additionally, young adults in Massachusetts reported easily accessing flavored products through social sources despite the FTR [43]. Another potential explanation is policy enforcement challenges. Because policy implementation coincided with the COVID-19 pandemic, there were disruptions and competing interests for public health officials. The quantity of seized illicit tobacco products increased each year after implementation, suggesting enforcement activities have ramped up since the first year [44]. If there were broader geographic restrictions on menthol cigarettes (e.g., a national menthol ban) that more widely reduced access to the product and had effective enforcement, perhaps the hypothesized effect would have been observed.

### Strengths and limitations

 This study has several strengths that should be noted. First, this study used a DID approach, which allows us to estimate causal effects of the policy using a pre-post analysis and a control state. Second, we looked at the policy’s impact both overall and among groups with higher rates of flavored tobacco use (African Americans and sexual minorities) to determine if the policy has differential impacts on specific populations. Finally, we used BRFSS data, giving us large, representative samples of the adult population in Massachusetts and Connecticut.

There are also several study limitations to note. First, there was only one post-policy timepoint and it was one year after the policy was implemented. Therefore, in addition to this being a short period to observe a behavior change such as cigarette cessation or decreased initiation, we were unable to look at trends after the policy was implemented. Second, the trends in tobacco use prior to policy implementation were not perfectly parallel between Massachusetts and Connecticut, suggesting the parallel trends assumption may not have been satisfied for all models; if there were other factors influencing tobacco use trends between 2019 and 2021, this may lead to biased results. Finally, categorization of sexual minorities was done in such a way that if a respondent provided an answer (not missing data or a refusal to answer) and did not explicitly say they were straight/heterosexual (they responded “don’t know/not sure”), they were classified for this analysis as a sexual minority. This was done to be as inclusive as possible; however, by categorizing sexual minorities this way, it is likely that some people who do not identify as a sexual minority were erroneously classified as such. This potential misclassification bias may result in an underestimation of the true effect size.

## Conclusions

This study evaluated the impact of Massachusetts’ FTR on adult cigarette use both overall and among African Americans and sexual minorities. Contrary to our original hypotheses, the policy did not lead to a greater reduction in tobacco use among adults overall, among African American adults, or among sexual minority adults in Massachusetts relative to a control state one year after policy implementation. There was some evidence that the policy had a detrimental impact on cigarette use among sexual minorities, but the reasons for this finding remain unclear. Future research should continue surveilling tobacco use trends in Massachusetts as well as other states with FTRs; examining trends overall and among marginalized groups is essential to understand if the policies are having an equitable effect. Additionally, qualitative research may be particularly useful to understand *why* the policy has not had the hypothesized impact. These data could help inform policy modifications, from adoption to enforcement, and further the goal of equitably reducing tobacco use.

## Supplementary Information

Below is the link to the electronic supplementary material.

Supplementary file1 (DOCX 17 KB)

## Data Availability

Code for the secondary data analysis is available upon request from the corresponding author.
