# Supplementary material for: An evaluation of Massachusetts’ flavored tobacco restriction one year after policy implementation: Is it promoting equity?
Source: J Public Health Policy. 2026 May 6;47(2):182–93. doi: 10.1057/s41271-026-00633-w (PMC13278950; doi:10.1057/s41271-026-00633-w)
Supplement: Supplementary file 1 — Supplementary file1 (DOCX 17 KB) [file 41271_2026_633_MOESM1_ESM.docx]

Supplementary Table 1. Ns and Weighted Percentages of current cigarette smoking among adults in Massachusetts and Connecticut, BRFSS Data 2015-2021

|  | Overall | | African American | | Sexual Minority | |
| --- | --- | --- | --- | --- | --- | --- |
|  | n | Weighted % | n | Weighted % | n | Weighted % |
| Massachusetts |  |  |  |  |  |  |
| 2015 | 1221/  8812 | 14.0% | 68/  472 | 15.7% | 79/  397 | 16.6% |
| 2016 | 1048/  8033 | 13.6% | 57/  401 | 11.3% | 70/  463 | 14.7% |
| 2017 | 767/  6723 | 13.7% | 34/  311 | 15.5% | 50/  332 | 19.1% |
| 2018 | 756/  6536 | 13.4% | 40/  323 | 10.9% | 65/  389 | 16.4% |
| 2019 | 828/  7507 | 12.1% | 48/  390 | 14.4% | 67/  416 | 15.7% |
| 2021 | 735/  7445 | 10.6% | 41/  412 | 10.9% | 81/  569 | 16.0% |
|  |  |  |  |  |  |  |
| Connecticut |  |  |  |  |  |  |
| 2015 | 1309/  11378 | 13.5% | 145/  930 | 16.9% | 85/  513 | 14.9% |
| 2016 | 1150/  10718 | 13.4% | 110/  643 | 16.8% | 68/  404 | 20.9% |
| 2017 | 1064/  10175 | 12.7% | 97/  634 | 15.0% | 67/  394 | 17.6% |
| 2018 | 1089/  10362 | 12.2% | 125/  732 | 18.2% | 91/  589 | 16.4% |
| 2019 | 892/  8738 | 12.1% | 88/  586 | 14.2% | 72/  538 | 14.8% |
| 2021 | 839/  7725 | 11.1% | 78/  507 | 11.9% | 80/  593 | 12.0% |
